# Flame Retardancy and Thermal Behavior of an Unsaturated Polyester Modified with Kaolinite–Urea Intercalation Complexes

**DOI:** 10.3390/molecules25204731

**Published:** 2020-10-15

**Authors:** Lina Yue, Junfei Li, Xuan Zhou, Yingjuan Sun, Ming Gao, Taohua Zhu, Xiaoqian Zhang, Teng Feng, Zhanhong Shi, Yongchun Liu

**Affiliations:** 1 Heibei Key Laboratory of Hazardous Chemicals Safety and Control Technology, School of Chemical and Environmental Engineering, North China Institute of Science and Technology, Yanjiao, Beijing 101601, China; yuelina@ncist.edu.cn (L.Y.); syj040520@ncist.edu.cn (Y.S.); gaoming@ncist.edu.cn (M.G.); zhangxiaoqian@ncist.edu.cn (X.Z.); fteng0013@163.com (T.F.); shizhanhong2020@163.com (Z.S.); 17333653645@163.com (Y.L.); 2 School of Materials Science and Engineering, University of Science and Technology Beijing, Beijing 100083, China; junfeili0304@163.com; 3 School of Electronic Science and Control Engineering, Institute of Disaster Prevention, Yanjiao, Beijing 101601, China; zhutaohua0812@163.com

**Keywords:** unsaturated polyester, kaolinite–urea intercalation complex, flame retardant, thermogravimetry

## Abstract

Organic modified kaolinite-urea intercalation complex (KUIC) was prepared using dimethyl sulfoxide (DMSO) as the precursor of kaolinite intercalation. Its structure was characterized by Fourier transform infrared (FTIR) and X-ray diffraction (XRD). Subsequently, as a synergistic agent, KUIC was combined with flame retardant ammonium polyphosphate (APP) to improve the flame retardant and smoke suppression performance of unsaturated polyester (UP) resin. A cone calorimeter (CONE) was used to study its flame retardancy and smoke suppression, and a scanning electron microscope (SEM) and thermogravimetry (TG) were used to study the micro morphology of the char and flame retardant mechanism. The results show that 12 phr of APP and 3 phr of KUIC were doped into UP to obtain a 28.0% limiting oxygen index (LOI) value. Compared with UP, the heat release rate and smoke production of UP/APP/KUIC composites were greatly decreased. Meanwhile, KUIC indeed enhanced the mechanical properties of UP.

## 1. Introduction

Unsaturated polyester (UP) resins are one of the most widely used types of composite resins, accounting for about 80% of all thermosetting resins. They have a good range of mechanical properties, corrosion resistance, low weight, and processibility [1,2,3], and are widely used as matrixes of composite materials for the transportation sector, building industry, and electrical industry, etc. [4]. However, because typical polyester resins are highly flammable and produce large amounts of smoke and toxic acids when burned, their industrial use is limited [5,6].

Therefore, in order to increase and develop their commercial application, the main need is to produce a fire retardant system, reduce fire hazard, and realize fire prevention [7]. Many flame retardants for UP have been found in the past few decades, such as halogenated additives, ammonium salts, zinc compounds, montmorillonite, alumina trihydrate (ATH), zinc stannates, and zinc borate/boric acid [8,9,10,11,12,13,14]. Although halogenated flame retardants have a good flame retardancy effect, they gradually withdraw from the flame retardancy stage because of their toxic gas production [15]. As a typical halogen-free flame retardant, ammonium polyphosphate (APP) has the best flame retardant effect [16,17,18]. In addition, compared to other halogen-free systems, APP requires lower loadings, ensuring a lower cost, better property retention, and excellent flow [19]. However, the poor compatibility between APP and polymer often leads to the decreased mechanical properties of the materials. In order to improve the above problems, a lot of researchers mainly focus on the following two aspects: the surface modification (or microencapsulation) of existing APP products and the development of an efficient synergistic agent.

Ammonium polyphosphate (APP) is a flame retardant based on phosphorous and ammonium salts. It is a halogen-free flame retardant which is modified and improved with time to comply with environmental issues and evolving regulations [16,17,18]. The ability of APP to suppress the generation of smoke is attributed to its unique mechanism of intumescence. However, the mechanical properties of most substrates are not good when ammonium polyphosphate is used alone, so it is often compounded with other components to reduce its influence on their mechanical properties.

Kaolinite is one of the most widely distributed clay minerals in nature. In recent years, kaolinite has been widely used in flame retardants and other fields due to its high refractoriness, good thermal insulation and a good chemical stability [20,21,22,23]. Kaolin is an inorganic silicon-containing flame retardant with a low cost and significant smoke suppression effect. When the polymer is burned, silica is formed to cover the char layer, which plays a double role of adiabatic and shielding. However, untreated kaolinite tends to aggregate in the polymer and is required to be flame retardant in large amounts [24]. Kaolinite can be inserted in dimethyl sulfoxide (DMSO), urea, amino silane, and other high-polarity molecules, resulting in an increase in the base spacing [25,26,27]. Meanwhile, nitrogen, silicon, sulfur, and other elements in these compounds have flame retardant effects, which can theoretically improve the flame retardant effects of kaolinite.

In this study, using DMSO as the precursor of kaolinite intercalation, we prepared kaolinite–urea intercalation complexes (KUIC) by replacing the DMSO with urea. Then, KUIC, acting as a synergistic agent, was combined with APP in the UP system to study its influence on the thermal stability of UPR and the effect of the flame retardant and smoke suppression. Its thermal behavior and burning behavior were characterized by TG, limiting oxygen index (LOI), cone calorimeter (CONE), and SEM.

## 2. Results and Discussion

### 2.1. Characterization of Kaolinite and KUIC

**Figure 1:**
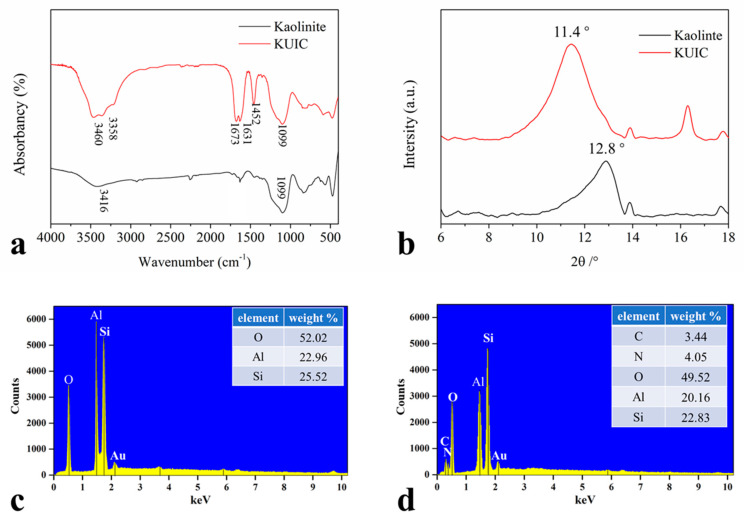
FTIR spectra (**a**) and XRD curves (**b**) of kaolinite and KUIC, and the elemental analyses of kaolinite (**c**) and KUIC (**d**).

The FTIR spectrum of parent clay kaolinite is shown in Figure 1a and Table 1. The absorptions at 3416 cm^−1^ in the -OH stretching region were attributed to the inner-surface -OH group [28]. Meanwhile, the major bands in the FTIR spectrum characterizing the KUIC molecule at 3460 and 3358 cm^−1^ in the -NH stretching region were attributed to the asymmetric and symmetric stretching vibrations of NH_2_ groups, which are hydrogen-bonded [29]. In addition, in the 1690–1400 cm^−1^ spectral range, three absorptions at 1673, 1631, and 1452 cm^−1^ can be observed. The vibration with the highest frequency is identified as the -C=O stretching vibration of urea. The next two bands belong to the deformation vibration of -N-H and the stretching vibration of -C-N. Si-O deformation bands are located in the 1200–1000 cm^−1^ region. In addition, the SEM-EDS spectrum also indicated that C and N elements were detected on the KUIC surface (see Figure 1c,d).

When organic molecules are inserted into the interlayers of kaolinite, the interlayer spacing of kaolinite will be more or less affected. By comparing the interlayer spacing of kaolinite before and after the insertion of small organic molecules, we can know whether organic molecules are inserted into the interlayers of kaolinite. From Figure 1b, the interlayer spacing of kaolinite original soil is 0.69 nm. When organic molecules are inserted into the interlayer of kaolinite, interlayer expansion is caused, and the interlayer spacing increases correspondingly. According to the FTIR and XRD results, DMSO was successfully substituted into the interlayer of kaolinite by urea, and the interlayer spacing of kaolinite was increased from 0.69 to 0.78 nm.

### 2.2. Flame Retardancy

**Figure 2:**
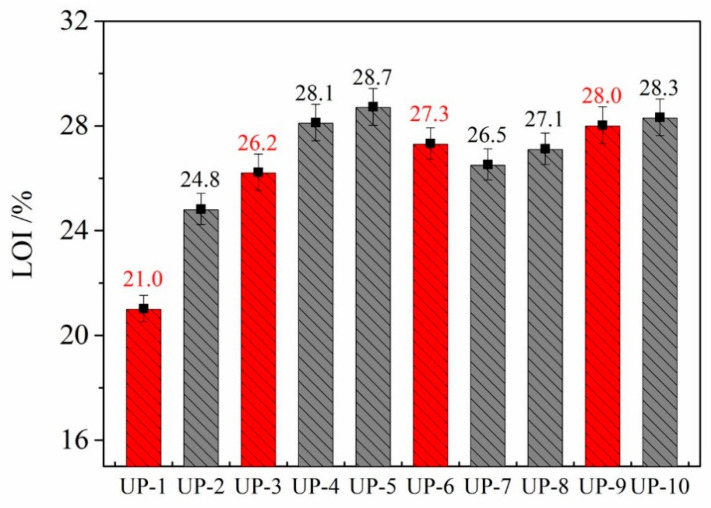
LOI values of all the UP samples.

Limiting oxygen index (LOI) is a fundamental tool in basic research on polymer combustion and the mechanism of flame retardancy. LOI also provides an evaluation of the intrinsic flammability of polymeric materials [30]. The flammability of UP samples is evaluated by determining their limiting oxygen indexes, and these values are presented in Table 2. The LOI value of neat UP is 21.0%, which increases from 24.8% to 28.7% as the APP content is increased from 10 to 25 phr, respectively, which shows the very good flame retardancy of APP. The LOI value of UP containing different amounts of APP is increased due to the presence of phosphorus and nitrogen in APP. Phosphorus and nitrogen have a synergistic effect between them, which enhances the LOI of UP. The trend of these data shows that the LOI increases with the increasing content of APP. Meanwhile, there is a similar trend of LOI values from 26.5% to 28.3%, with the increasing content of KUIC from 1.0 to 4.0 phr, though the increases are much little, which is due to the limited flame retardancy of KUIC, but this is better than that of kaolinite because of the higher LOI for UP-9 of 28.3% than for UP-6 of 27.3%. The results of the UL 94 test showed that the combustion rating of the composites containing flame retardant and clay (kaolinite or KUIC) reached a V0 rating and V1 rating. When 15% APP was added, the UP composites reached a V0 rating, and the addition of clay (kaolinite and KUIC) had no effect on the combustion rating of the UP composites; all reached the V0 rating (shown in Figure 2).

The synergistic effectivity (SE) and flame retardant effectivity (EFF) are often used to evaluate the synergistic flame retardant effect of a system. EFF is defined as the increase rate of the oxygen index for every 1% increase in the flame retardant effects; SE is defined as the ratio of the flame retardancy with and without synergistic agents. The corresponding calculation formulas are as follows:

(1) $$\mathrm{EFF}=\frac{\Delta (\mathrm{LOI})}{\omega (\mathrm{APP})+\omega (\mathrm{Kaolinite}\;\mathrm{or}\;\mathrm{KUIC})}$$

(2) $$\mathrm{SE}\;=\;\frac{\mathrm{EFF}*}{\mathrm{EFF}0}$$

where “*” is the sample for UP/APP/kaolinite or UP/APP/KUIC, and “0” is the sample for the UP/APP. When SE > 1, APP and clay (kaolinite or KUIC) have a synergistic effect on the flame retardancy; the greater the SE value, the better the synergistic flame retardant efficiency. The calculation results of EFF and SE are summarized in Table 2. It can be seen that, with the gradual addition of KUIC, the EFF value and SE value show a trend of increase, and the synergistic efficiency of APP and KUIC is greater than 1, which shows that APP and KUIC have synergistic flame retardant effects.

### 2.3. Mechanical Properties

**Figure 3:**
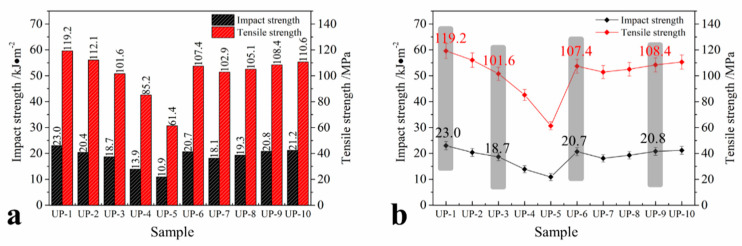
Impact strength (**a**) and tensile strength (**b**) of the UP samples.

The mechanical properties of UP are important parameters that determine its applications, such as in load bearing and as packaging materials. Tensile strength and impact strength are very important parameters corresponding to mechanical properties. To study the effect of the APP and KUIC loading on the mechanical properties of UP, the tensile strength and impact strength of UP containing different APP and KUIC loadings were determined and are shown in Figure 3. These indicate that the tensile strength and impact strength of UP decreased with increases in the APP loading. This decrease in the mechanical properties was due to its poor wetting or adhesion with the polymer matrix; this also, with the inclusion of higher amounts, leads to agglomeration, because the filler–filler interaction becomes more pronounced. However, the decreases in the mechanical properties are less than 20% with lower loadings of APP (below 15 phr)—only 19% for the tensile strength and 15% for the impact strength for UP-3 containing 15 phr of APP, which has good flame retardancy with a LOI of 26.2%.

On the other hand, from Figure 3 the tensile strength and impact strength increased with increases in the KUIC loading, and there is no obvious difference between kaolinite and KUIC. The enhancements are mainly attributed to the goof dispersion and high exfoliation of the silicate layers in the UP matrix, which provided high resistance against the plastic deformation and the effect of the stretching of the oriented backbone bonds of the polymer chains in the gallery [31].

### 2.4. Cone Calorimeter Test

**Figure 4:**
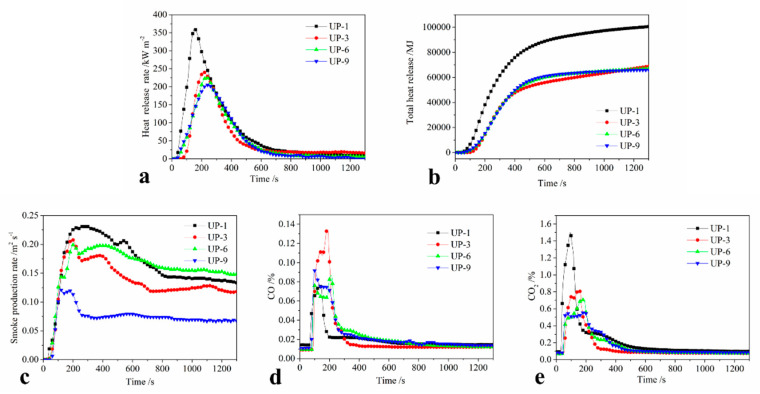
The cone calorimeter test curves of UP samples: (**a**) heat release rate, (**b**) total release rate, (**c**) smoke production rate, (**d**) CO yield, and (**e**) CO_2_ yield.

The cone calorimeter was an ideal instrument to evaluate realistically the combined flame-retarding and smoke-suppressing behavior of polymer. Some cone calorimeter results have been found to correlate well with those obtained from large-scale fire tests and can be used to predict the behavior of materials in real fires. Some of the important parameters of PU-1, PU-3, PU-6, and PU-9 derived from the cone calorimeter are shown in Figure 4 and Table 3.

Figure 4a shows the heat release rate (HRR) curves as functions of the combustion time of all the samples. The HRR and peak HRR decreased markedly when UP is composited with APP, kaolinite, and KUIC. From Figure 4a and Table 3, it can be seen that the peak HRR of UP-3 is 240.7 kW·m^−2^, which decreased by 33.4% compared with UP-1 (361.2 kW·m^−2^). This is because APP can promote charring to form carbonaceous materials, which can insulate oxygen and heat during combustion. For UP-6, the peak HRR is 225.9 kW·m^−2^, lower than that of UP-3, which may be due to the fact that kaolinite improves the thermal stabilities of UP, as mentioned above. The peak HRR is 205.4 kW·m^−2^ for UP-9, which decreased by 43% compared with UP-1. This is lowest peak HRR in all the samples, which may be due to the fact that the KUIC can greatly increase the thermal stabilities of UP or probably promote the UP product with much more compact and continuous char yields because of the synergistic effect between APP and KUIC. Meanwhile, at 1200 s the total heat release (THR) of the UP-3, UP-6, and UP-9 samples was lower than that of the UP-1 sample. As a result, the THR of UP-9 declined from 103751 MJ of pure UPR to 66114 MJ and reduced by 36.3%. It was concluded that the addition of APP and KUIC effectively constrained the heat release of the UP thermosets.

Generally, the CO_2_ release from materials is closely related to their inflammability; the more CO_2_ there is, the more the inflammability there is. Meanwhile, the CO productions are due to the incomplete combustion of the materials. Figure 4d,e show the CO_2_ and CO content in the gas products from the samples, respectively, and the corresponding data are shown in Table 3. The CO_2_ release of pure UP is more and faster than that of other samples, which indicates that UP sharply burns and completes in 600 s. The CO_2_ content is significantly decreased for other samples in the burning products. The CO_2_ content of PU-3, PU-6, and PU-9 is 0.81%, 0.71%, and 0.55%, respectively, which indicates their different flame retardancy, which is better for PU-3 and PU-6 and best for PU-9, and is in agreement with the results of HRR and LOI. A similar rule is shown for the CO release as in Figure 4d. From Figure 4e, we can see that the CO release of the flame retardant UP (PU-3, PU-6, PU-9) is more than that of the pure UP sample, since it means the incomplete combustion of the materials. Moreover, the CO content of PU-9 is less than that of PU-3 and PU-6, which is very favorable for its practical application with the best flame retardancy and the lowest toxicity.

Meanwhile, the smoke production along with the heat release rate plays a critical role in fire conditions. Figure 4c and Table 3 show that the peak smoke production rate (SPR) of samples decreased to different degrees after adding the flame retardant. The total smoke production (TSP) of UP-6 decreased from 190.2 m^2^·kg^−1^ (UP-3) to 161.2 m^2^·kg^−1^ after the addition of kaolinite, which shows that kaolin can increase the stability of the carbon layer and inhibit the emission of smoke. With the presence of 15 phr APP and 3 phr KUIC, the smoke suppression of the UP-9 sample is more prominent, and the TSP of UP-9 (121.7 m^2^·kg^−1^) decreases by 38.4% compared with that of pure UP (197.5 m^2^·kg^−1^). KUIC can promote the thermal decomposition of UPR to form a denser carbon layer and inhibit the combustion of UPR, thus slowing down the heat release rate of UPR and reducing the total amount of smoke.

### 2.5. Thermal Degradation

When flame retardants are incorporated into polymeric materials, the weight loss pattern of polymers is altered. Phosphorus-nitrogen groups decompose at relatively lower temperatures to form a heat-resistant char, retarding the weight loss rate of the polymers at higher temperatures through condensed-phase mechanisms [32].

**Figure 5:**
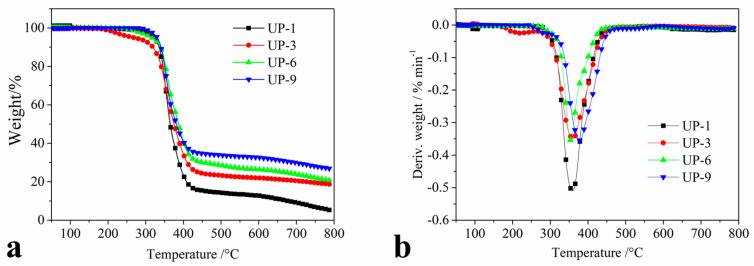
(**a**) TGA and (**b**) DTG curves of the UP samples under nitrogen.

The measurement of the TG and DTG curves of UP-1, UP-3, UP-6, and UP-9 was carried out in dynamic air from an ambient temperature to 800 °C, and they are shown in Figure 5. The initial decomposition temperature (IDT) was determined by a 5% weight loss, showing the samples beginning to degrade, and indicates the apparent thermal stability of the samples. The integral procedure decomposition temperature (IPDT) was determined by a 50% weight loss, and exhibits the samples’ inherent thermal stability. The weight loss (WL) at main decomposition, the char yield (CY) at 700 °C, the temperatures at the maximum weight loss rate (*T*_max_), and the value of the maximum weight loss rate (*R*_max_) were measured and are listed in Table 4.

The results indicate that neat UP is thermally stable below 300 °C, and IDT is 321 °C. It has a small amount of volatile until the temperature rises to 340 °C, and it has a weight loss of less than 20% at 340 °C. When the temperature further increases, the weight loss increases rapidly and a quantity of volatile is produced until it almost exhausts at 420 °C, and the char yields at 700 °C are 9.1%. The TG and DTG curves of UP/APP (UP-3) are different. It is noted that IDT (252 °C) and *R*_max_ (0.35 %/min) are decreased while the char yields (20.6%) are increased. This is because the APP in UP-3 begins to decompose at relative low temperatures, generating some small molecules such as phosphoric acid, water, ammonia, etc. [33]. With the increase in temperature, it is observed that APP can increase the number of residues remarkably, which means that APP promotes charring to form carbonaceous materials. This is one of the reasons for the improvement in the flame retardancy of UP. *R*_max_ is decreased in the UP/APP/kaolinite (UP-6), and its char yields are increased. Its IDT is increased compared with that of UP-3 and UP, which indicates the kaolinite can improve the thermal stabilities of UP. UP/APP/KUIC (UP-9) has similar thermal behaviors to those of UP/APP, and *R*_max_ (0.36%/min) is decreased, while the char yields (24.5%) are remarkably increased. Meanwhile, its IDT (310 °C) is increased, which shows that the thermal stabilities of UP are improved by the incorporation of KUIC. It is possible that KUIC can form an effective charring layer in lower decomposition, preventing the char layer from oxidation, and getting good flame retardancy due to the synergistic effect between APP and KUIC. The higher LOI values in Table 2 support this notion.

The thermal stability of the UP is assessed by two parameters: IDT and IPDT. IDT indicates the apparent thermal stability of the UP resins—i.e., the failure temperatures of the resins in processing and moulding. On the other hand, IPDT exhibits the inherent thermal stability of the resins, i.e., the decomposition characteristics of the volatile composition. From Table 4, flame retardant UP (UP-3, UP-6, UP-9) show relatively lower IDTs than do the neat resin (UP-1), since APP decomposes at low temperatures. On the other hand, the existence of kaolinite (in UP-6) and KUIC (in UP-9) exhibits higher IPDT than the UP containing only APP (UP-3), exhibiting a higher thermal stability, especially for KUIC.

### 2.6. Morphology of Residual Char

**Figure 6:**
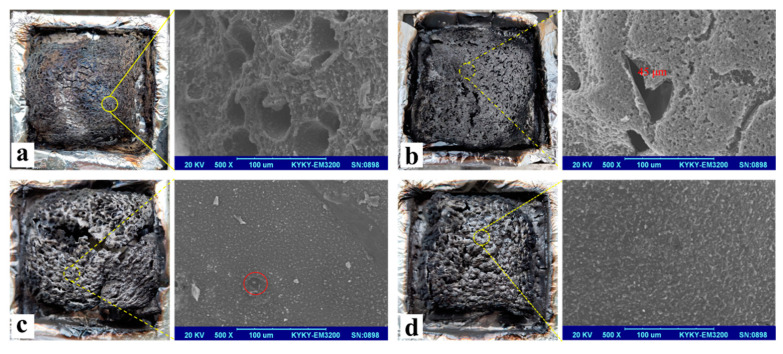
Digital images and SEM micrographs of the residual char from the combustion of UP-1 (**a**), UP-3 (**b**), UP-6 (**c**), and UP-9 (**d**) after the cone calorimeter test.

It is well known that an effective protective char layer could improve the flame retardancy of materials during combustion. The TG results indicate that the char yields of UP-3, UP-6, and UP-9 are increased by the incorporation of APP, kaolinite, and KUIC. This is necessary but not sufficient to ensure fire protection, because the char formed may be too light and not sufficiently mechanically resistant. A structure with a relatively strong char layer is important to minimize the heat transfer and provide good protection for the substrate [34]. Thus, the morphologies of the residual char were investigated by SEM. Figure 6 shows the SEM micrographs of the residual char from the combustion of UP-1, UP-3, UP-6, and UP-9 after the CONE test. As can be seen from Figure 6a, it is obvious that loose and flaky char is observed at the outer surface of UP, with many large holes scattered on the surface which could not protect the underlying material from fire. From Figure 6b, we can see that a continuous char is formed in the interior part, which corresponds to the condensed-phase mechanisms of APP. However, there are a few holes or bubbles on the surface. However, due to the presence of clay, the surface continuity of the condensed char layer of UP-6 is worse than that of UP-3, as shown in Figure 6c. Meanwhile, the char residue of UP-9 depicted in Figure 6d exhibits a much more compact and continuous morphology compared with that of the other UP samples. A honeycomb-like structure is obtained within the residual char, indicating the formation of a strong charred layer during combustion. It has been reported [35] that honeycomb-like char probably exhibits much better heat insulation and mechanical properties. Based on the SEM result, it is reasonable to believe that the formation of a cohesive and dense structure of residual char is responsible for the thermal stability and flammability properties. The formation of efficient char for UP-9 can prevent heat transfer between the flame zone and the substrate, and thus protect the underlying materials from further burning and pyrolysis.

### 2.7. Flame Retardant Mechanism

**Figure 7:**
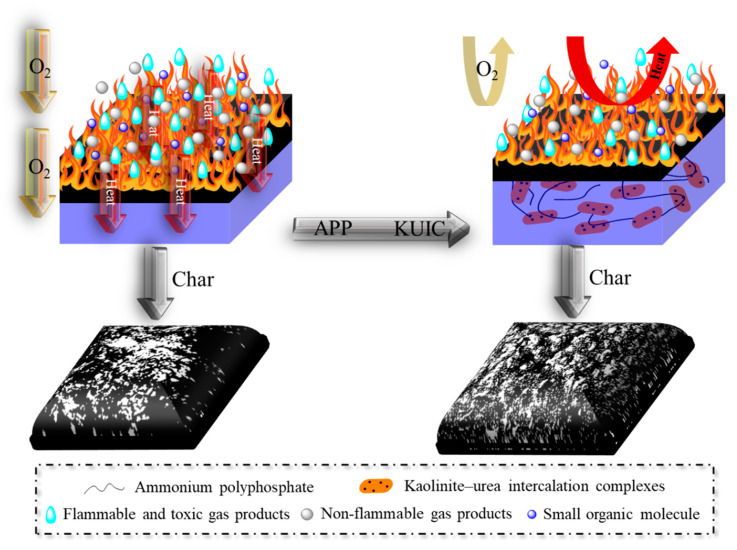
Proposed flame retardant mechanism of the UP/APP/KUIC samples.

Combined with the before-mentioned analysis and some previous literature [36,37,38], a possible flame retardant mechanism of the UP/APP/KUIC composites is proposed in Figure 7. In the pure sample exposed to a heat source, the resulting carbon layer was unable to isolate oxygen and heat transfer, causing the polymer to burn violently. However, when exposed to a radiation heat source, phosphoric acid and metaphosphoric acid are released by APP, and the obtained acid can catalyze the intramolecular or intermolecular dehydration of UP composites to form cokes. The resulting cross-linked cokes play a very good role in thermal insulation and oxygen shielding. In addition, APP is decomposed into a lot of none-flammable gas products (NH_3_, H_2_O), which reduces the concentration of flammable gases and oxygen in the gas phase, thus preventing further burning of the base material. Meanwhile, a lot of phosphorus free radicals (HPO•, PO_2_•, PO• and HPO_2_•) are produced during combustion, which capture H• and OH• free radicals [38]. In here, the KUIC nanomaterials further promote the UP/APP system to form a dense coke layer at the interface between the gas phase and the condensed phase, acting as a barrier for heat exchange and mass exchange. The introduction of urea between the kaolin layers not only improved the dispersion of clay, but also showed a good synergistic effect with the APP. The char network formed by KUIC and crosslinked polyphosphoric acid can be strengthened, making the char more stable, and acts as an oxygen-insulating and heat-insulating function, ultimately achieving flame retardancy.

## 3. Materials and Methods

### 3.1. Materials

Orthophthalic unsaturated polyester resin (UP, type:191#, commercial grade), curing agent methyl ethyl ketone peroxide (MEKP), and accelerant agent cobalt naphthenate were purchased from Central Henan Yizhiyuan chemical products Co., LTD. Dimethyl sulfoxide (DMSO, 99.8%, AR) and urea (99%, AR) were supplied by Tianjin Fuchen chemical Reagent Co., Ltd. (Tianjin, China), and kaolinite (98%, CP) was obtained from Tianjin Zhiyuan chemical reagent Co., Ltd. (Tianjin, China). APP was bought from the Tangshan Yongfa flame retardant material factory (Tangshan, China).

### 3.2. Preparation KUIC of UP/APP/KUIC Composites

**Figure 8:**
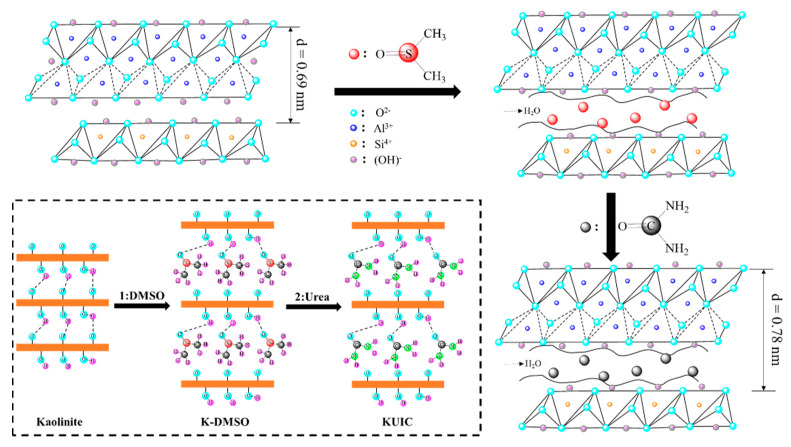
Preparation process of the kaolinite–urea intercalation complex.

KUIC is prepared by intercalating dimethyl sulfoxide with kaolin and then replacing it with urea [39]; a composite schematic diagram of KUIC is shown in Figure 8. The formulation of the composites mainly included polyester, curing agent, accelerator, APP, and KUIC. Composite sample formulations were prepared by adding different amounts of KUIC to UP. The formulation of the composites prepared was specified in the Table 5. The UP, curing agent, and accelerator were initially mixed and then APP and KUIC were added and mixed for about 5 min. Then, the mixture was poured into aluminum molds. The composites were cured for 20 min at 25 °C and post cured at 50 °C for 5 h.

### 3.3. Testing

The characterizations of all samples by X-ray diffraction (XRD) were carried out with an X-ray diffractometer (RAD-3C, Rigaku, Japan). We used Cu Kα radiation (Kα = 1.54 Å) operating at 40 kV and 40 mA. The scanning rate was 2°/min. Fourier transform infrared (FTIR) spectroscopy analyses were performed with an FTS 2000 FTIR (Varian, Ok, USA). We used this spectrometer in the FTIR range of 4000–400 cm^−1^. The limiting oxygen index (LOI) test was performed with a JF-3 oxygen index test instrument (Jiangning, China) in terms of the standard LOI test, ASTM D 2863-97. The specimen size for the LOI measurement was 130 × 6.5 × 3 mm^3^. The UL 94 rating test used a PX03001-02 vertical combustion tester (PHINIX, jiangsu China) according to GB/T 5169 (sample dimension: 3.2 mm × 12.7 mm × 127 mm). Thermogravimetry (TG) was carried out on an HCT-2 thermal analyzer (Beijing Hengjiu Scientific Instrument Factory) under a dynamic air (dried) atmosphere at a heating rate of 10 °C min^−1^ from room temperature to 700 °C. The specimen size for the cone calorimetry experiments was 100 × 100 × 3 mm^3^ by PX-07-007 (Phoenix quality inspection instrument co., LTD, Shanghai, China). The char residues after the CONE test were studied with a scanning electron microscope (KYKY-EM3200, China). The samples were gold-coated before scanning to provide an electrically conductive surface. An accelerating voltage of 20 kV was used while we recorded the scanning electron micrograms. The elemental analyses of kaolinite and KUIC were studied by an energy-dispersive X-ray spectrometer (EDS, Ns-7 Thermo Fisher Scientific, Waltham, MA, USA) equipped with a scanning electron microscope (SEM, KYKY-EM3200, Beijing, China).

## 4. Conclusions

In this study, the liquid phase interdressing method was used to insert DMSO into kaolin to obtain a K-DMSO precursor, and then replace DMSO with urea to obtain KUIC. UP/APP/KUIC composites were prepared, in which APP enhances the flame retardancy and KUIC improves the thermal properties of the UP resin. A total of 15 phr of APP and 3 phr of KUIC were doped into UP to obtain a 28.0% LOI, in which KUIC has a better flame retardant efficiency than kaolinite. Compared with UP, the THR and TSP decreased reduced from 10.0 × 10^4^ MJ and 197.5 m^2^·kg^−1^ for the control UPR sample to 6.6 × 10^4^ MJ and 121.7 m^2^·kg^−1^ for the UP/APP/KUIC composites, respectively. The tensile strength and impact strength of UP decreased with increases in the APP loading, while it increased with the increase in kaolinite and KUIC.

The APP in UP/APP/KUIC composites began to decompose at relatively low temperatures, which could catalyze the decomposition and carbonization of UP to form carbonaceous materials, leading to a low thermal stability and *R*_max_ and less weight loss. Meanwhile, the KUIC in the UP/APP/KUIC composites increased the thermal stability and char yields. Ultimately, a residual char with a honeycomb-like structure is obtained with a better heat insulation and mechanical properties during the combustion of the UP/APP/KUIC composites by the presence of 12 phr of APP and 3 phr of KUIC, which could protect the underlying materials from further burning and pyrolysis and achieve a good flame retardancy.

## Figures and Tables

**Table 1:** Characteristic bands of kaolinite and KUIC.

Kaolinite/cm^−1^	KUIC/cm^−1^	FTIR of Vibration Properties
-	3460	symmetric stretching modes of -N-H
3416		stretching mode of -O-H
-	3358	asymmetric stretching modes of -N-H
	1673	stretching mode of -C=O
-	1631	deformation vibration of -N-H
-	1452	stretching vibration of -C-N
1099	1099	deformation vibration of -Si-O

**Table 2:** Data of the LOI, EFF, and SE of UP and its composites.

Sample	ω_(APP)_/%	ω_(Kaolinite)_/%	ω_(KUIC)_/%	LOI/%	Δ_(LOI)_/%	EFF	SE
UP-1	-	-	-	21.0	-	-	-
UP-3	15	-	-	26.2	5.2	0.35	-
UP-6	12	3	-	27.3	6.3	0.42	1.20
UP-7	14	-	1	26.5	5.5	0.37	1.06
UP-8	13	-	2	27.1	6.1	0.41	1.17
UP-9	12	-	3	28.0	7.0	0.47	1.34
UP-10	11	-	4	28.3	7.3	0.49	1.40

**Table 3:** The cone calorimeter test data of the UP samples.

	UP-1	UP-3	UP-6	UP-9
peak HRR/kW·m^−2^	361.2 ± 1.6	240.7 ± 3.7	225.9 ± 2.5	205.4 ± 1.9
THR/MJ	103751 ± 45	69125 ± 23	67178 ± 51	66114 ± 37
peak SPR/m^2^·s^−1^	0.23 ± 0.06	0.21 ± 0.04	0.20 ± 0.05	0.12 ± 0.03
TSP/m^2^·kg^−1^	197.5 ± 4.1	190.2 ± 3.4	161.2 ± 1.7	121.7 ± 2.1
CO yield/%	0.07 ± 0.01	0.13 ± 0.03	0.08 ± 0.02	0.09 ± 0.03
CO_2_ yield/%	1.50 ± 0.21	0.81 ± 015	0.71 ± 0.11	0.55 ± 0.10

**Table 4:** Thermal data of the UP samples from the thermogravimetric analysis.

No.	IDT /°C	IPDT /°C	WL /%	CY /%	*T*_max_ /°C	*R*_max_ %/min
UP-1	321	364	78.0	9.1	359	0.51
UP-3	252	375	69.1	20.6	359	0.35
UP-6	305	380	65.6	22.1	351	0.33
UP-9	310	386	62.8	24.5	378	0.36

**Table 5:** Composition of the UP samples.

No.	UP/phr	Curing agent/phr	Accelerator/phr	APP/phr	Kaolinite/phr	KUIC/phr
UP-1	94	3	3	-	-	-
UP-2	84.6	2.7	2.7	10	-	-
UP-3	79.9	2.55	2.55	15	-	-
UP-4	75.2	2.4	2.4	20	-	-
UP-5	70.5	2.25	2.25	25	-	-
UP-6	79.9	2.55	2.55	12	3	-
UP-7	79.9	2.55	2.55	14	-	1
UP-8	79.9	2.55	2.55	13	-	2
UP-9	79.9	2.55	2.55	12	-	3
UP-10	79.9	2.55	2.55	11	-	4
